# Editorial: The link between metabolic syndrome and chronic kidney disease: focus on diagnosis and therapeutics - volume II

**DOI:** 10.3389/fendo.2024.1442803

**Published:** 2024-06-13

**Authors:** Kexin Zhang, Weixia Sun, Guiting Lin, Ningning Hou

**Affiliations:** ^1^ Department of Endocrinology and Metabolism, Clinical Research Center, Shandong Provincial Key Medical and Health Discipline of Endocrinology, Affiliated Hospital of Shandong Second Medical University, Weifang, China; ^2^ Department of Nephrology, the First Hospital of Jilin University, Jilin, China; ^3^ Knuppe Molecular Urology Laboratory, Department of Urology, University of California, San Francisco, San Francisco, CA, United States

**Keywords:** metabolic syndrome, obesity, chronic kidney disease, diagnosis, therapeutics

Metabolic syndrome (MS), characterized by obesity, hyperglycemia, hyperuricemia, etc., is intricately associated with the development of chronic kidney disease (CKD) (1–3). Understanding this association has become paramount due to the rising prevalence of MS. However, the diverse pathophysiology of kidney injury stemming from various metabolic risk factors pose challenges for accurate diagnosis and effective therapy. Investigating the interactions between MS-related ailments and CKD is crucial for enhancing diagnostic and therapeutic approaches. This Research Topic provides a comprehensive platform for presenting recent advancements in diagnosing and treating multiple MS-related CKD, comprising a total of 14 articles, including 10 original research articles, 3 review articles, and 1 hypothesis and theory article.

One significant area of interest lies in identifying biomarkers heralding CKD onset or progression in individuals with MS. The Triglyceride-glucose (TyG) index emerges as a calculated measure used in clinical research to assess insulin resistance. Wang et al. underscore the significance of the TyG index as a valuable marker for assessing metabolic dysfunction and its association with albuminuria in the adult population of the United States. They found that elevated TyG index levels were independently linked to albuminuria, demonstrating its superiority over traditional indicators such as insulin resistance in predicting albuminuria. Similarly, Dong et al. highlight the significant association between the TyG index and early renal impairment in hypertensive patients. Elevated TyG index levels correlate with increased serum levels of β2-microglobulin and cystatin C, suggesting a role in renal dysfunction progression. However, caution is advised in using the TyG index alone as a predictor since it falls short of surpassing traditional markers like triglycerides in predicting early renal impairment. Further research is needed to clarify its utility and limitations in assessing renal health, highlighting the need for further investigation.

In addition, lipid metabolism emerges as pivotal in CKD progression, with Tian et al. presenting a nomogram model integrating low-density lipoprotein cholesterol levels for precise prognostication in IgA nephropathy. The association between remnant cholesterol (RC) levels and CKD in hypertensive patients, as elucidated by Yuan et al., further underscores the importance of lipid management in CKD prevention and treatment. They found a linear positive relationship between RC and CKD risk. Subgroup analysis further indicated a more pronounced association among patients with a BMI ≥24 kg/m^2^ and those who were current non-smokers. Their findings provide valuable insights into the modifiable risk factors for CKD in high-risk populations, informing targeted interventions and personalized management strategies.

Identifying new biomarkers for early CKD diagnosis is crucial, especially in newly diagnosed patients with different glycemic statuses. Cassano et al. discovered that elevated 1-hour post-load glucose levels (≥155 mg/dl) are linked to higher CKD risk in these patients. They also observed escalating oxidative stress, platelet activation, and deteriorating metabolic profiles in early CKD. Moreover, the exploration of novel markers such as METS-VF for kidney stone risk assessment, as investigated by Guo et al., highlights the multifaceted nature of MS-related renal complications. Research utilizing NHANES data investigated METS-VF’s correlation with kidney stones in 29,246 participants. Results uncovered a positive correlation between METS-VF and kidney stone prevalence and progression. These findings underscore the potential of METS-VF as a marker for assessing kidney stone risk and emphasize the crucial role of addressing visceral fat in preventive strategies.

Diagnostic innovations also take center stage, with Cao et al. leveraging bioinformatics analysis and machine learning to identify biomarkers for CKD in patients with non-alcoholic fatty liver disease (NAFLD). The study identifies four diagnostic markers (DUSP1, NR4A1, FOSB, ZFP36) enriched in immune-related pathways and inflammatory responses, showing promising diagnostic utility in CKD patients with NAFLD. Moreover, innovative imaging techniques, such as mDIXON-Quant, offer promise for early CKD diagnosis and assessment of renal damage severity. Wang et al.‘s retrospective study underscores the clinical promise of mDIXON-Quant imaging for early CKD detection and assessing renal damage severity, fostering enhanced patient stratification and management approaches.

Advancements in biomarker diagnosis for CKD are paving the way for personalized early detection and treatment strategies. Tian et al.‘s cross-sectional study underscores serum uric acid (SUA) levels as an independent risk factor for CKD in adolescents, advocating for early monitoring and intervention, especially across diverse BMI populations. They found a negative association between SUA and eGFR, notably among adolescents aged 12–19 years. Higher BMI correlated with elevated SUA, impacting eGFR, particularly in underweight adolescents. Tailored CKD prevention and management strategies in adolescence are warranted based on these findings.

Optimal treatment for CKD remains a significant challenge beyond diagnosis. Emerging therapeutic modalities offer hope for improved outcomes in patients with MS-related CKD. Lv et al. ‘s review on finerenone underscores its potential in mitigating cardiovascular and renal complications in type 2 diabetes patients with CKD. As a third-generation mineralocorticoid receptor antagonist, finerenone offers improved safety and efficacy, potentially benefiting not only cardiovascular and renal health but also conditions like diabetic kidney disease (DKD). The heightened selectivity and efficacy of finerenone present a promising avenue for addressing the intricate web of metabolic and renal dysfunction in these patients.

Vasopressin, known as a stress hormone, influences kidney function and metabolism through various receptor types. Lebedeva et al. explore how elevated plasma vasopressin levels contribute to the pathophysiology of DKD. Excessive activation of renal V2 receptors leads to glomerular hyperfiltration, while stimulation of extra-renal V1a/V1b receptors worsens DKD by promoting catabolic metabolism. Selective vasopressin receptor antagonists show promise in separating renal and extra-renal effects, offering potential for therapeutic development. Understanding these mechanisms is vital for advancing future treatment strategies for DKD.

Dong et al. identified immune-related genes (IRGs) with potential as therapeutic targets for renal interstitial fibrosis (RIF) in CKD. They pinpointed 17 IRGs associated with immune response, highlighting six key ones: apolipoprotein H, epidermal growth factor, lactotransferrin, lysozyme, phospholipid transfer protein, and secretory leukocyte peptidase inhibitor. Further analysis revealed these IRGs’ connections to T cell populations and the NF-κB signaling pathway, suggesting their promise for RIF treatment in CKD.

Han et al.‘s review highlights autophagy as a promising therapeutic target in DKD. Dysregulated autophagy, influenced by nutrient-sensing and stress pathways such as AMPK, mTOR, and oxidative stress, drives DKD progression by disrupting cellular homeostasis. Targeted interventions aimed at restoring autophagic flux hold promise for mitigating renal dysfunction in DKD, offering hope for improved patient outcomes in addressing this pressing public health concern.

Zhang et al. conducted a thorough analysis of the genetic landscape of DKD complicated with inflammatory bowel disease IBD. They identified 495 risk genes, with MMP2, HGF, FGF2, IL-18, IL-13, and CCL5 emerging as key players. These genes are involved in inflammatory responses, oxidative stress, and immune dysfunction, suggesting potential targets for personalized treatments.

In summary, this research topic signifies a notable advancement in elucidating the complex interplay between MS and CKD. From novel biomarkers and therapeutic targets to innovative diagnostic modalities, the contributions within this collection pave the way for improved patient outcomes and personalized management strategies in this complex and multifaceted disease paradigm. Collaboration across disciplines and concerted research efforts will be essential in translating these findings into clinical practice and improving patient lives worldwide.

## Author contributions

KZ: Conceptualization, Investigation, Writing – review & editing, Formal analysis, Methodology, Writing – original draft. WS: Conceptualization, Investigation, Methodology, Writing – original draft. GL: Conceptualization, Writing – review & editing, Data curation, Project administration, Supervision, Validation. NH: Conceptualization, Investigation, Supervision, Validation, Writing – review & editing.
